# Role of human glutathione transferases in biotransformation of the nitric oxide prodrug JS-K

**DOI:** 10.1038/s41598-021-00327-1

**Published:** 2021-10-21

**Authors:** Birgitta Sjödin, Bengt Mannervik

**Affiliations:** Department of Biochemistry and Biophysics, Arrhenius Laboratories, Stockholm University, SE-10691 Stockholm, Sweden

**Keywords:** Biochemistry, Cancer

## Abstract

Nitric oxide (NO) plays a prominent physiological role as a low-molecular-mass signal molecule involved in diverse biological functions. Great attention has been directed to pharmacologically modulating the release of NO for various therapeutic applications. We have focused on O^2^-(2,4-dinitrophenyl) 1-[(4-ethoxycarbonyl)piperazin-1-yl]diazen-1-ium-1,2-diolate (JS-K) as an example of diazeniumdiolate prodrugs with potential for cancer chemotherapy. JS-K is reportedly activated by glutathione conjugation by glutathione transferase (GST), but the scope of activities among the numerous members of the GSTome is unknown. We demonstrate that all human GSTs tested except GST T1-1 are active with JS-K as a substrate, but their specific activities are notably spanning a > 100-fold range. The most effective enzyme was the mu class member GST M2-2 with a specific activity of 273 ± 5 µmol min^−1^ mg^−1^ and the kinetic parameters Km 63 µM, k_cat_ 353 s^−1^, k_cat_/Km 6 × 10^6^ M^−1^ s^−1^. The abundance of the GSTs as an ensemble and their high catalytic efficiency indicate that release of NO occurs rapidly in normal tissues such that this influence must be considered in clarification of the tumor-killing effect of JS-K.

## Introduction

**Figure 1:**
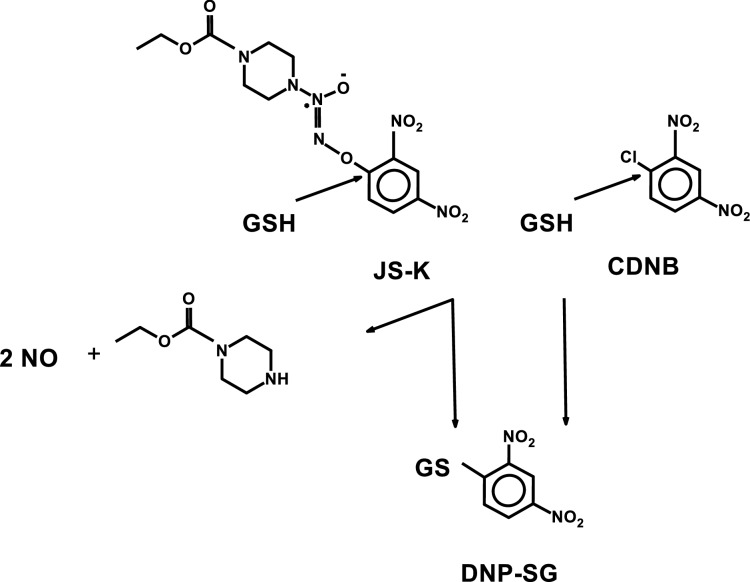
Structural similarities between JS-K (O^2^-(2,4-dinitrophenyl) 1-[(4-ethoxycarbonyl)piperazin-1-yl]diazen-1-ium-1,2-diolate) and CDNB (1-chloro-2,4-dinitrobenzene) and their conjugation with glutathione (GSH). The arrows show the site of nucleophilic attack by GSH leading to the common product S-2,4-dinitrophenylglutathione (DNP-SG), which can be monitored spectrophotometrically at 340 nm. JS-K yields also a second product (not shown), which decomposes into nitric oxide and 4-carboethylpiperazine.

The significant physiological role of gasotransmitters, such as nitric oxide (NO), hydrogen sulfide (H_2_S), and carbon monoxide (CO), has been unraveled by numerous investigations^1^. NO, most extensively, has been shown to serve as a mediator in various biological processes. More recently, attention to the signaling functions of H_2_S has risen to similar distinction and development of chemical derivatives that could serve as molecular vehicles for delivery of these gasotransmitters for pharmacological interventions is in progress^2, 3^. Among several NO-releasing prodrugs O^2^-(2,4-dinitrophenyl) 1-[(4-ethoxycarbonyl)piperazin-1-yl]diazen-1-ium-1,2-diolate (JS-K)^4^ has undergone trials in several animals models and shown promise as a novel prodrug in cancer chemotherapy^5^. Recent reports address approaches to overcome solubility issues and means to administer JS-K in order to improve its efficacy in targeted tissues^6, 7^. Common to JS-K and several related NO-releasing agents is their activation by glutathione (Fig. 1), which is promoted by soluble glutathione transferases (GSTs)^8^. The mammalian enzymes occur in seven classes^9^ of which members of the pi, mu, and alpha classes are major contributors to biotransformation of xenobiotics^10^. Some of these GSTs are overexpressed in cancer cells^11, 12^, suggesting that tumors could be selectively vulnerable and be targeted with prodrugs activated by the overexpressed enzyme. In general, the pi class member GST P1-1 has been the primary focus in this approach^13, 14^.

Since the original publication on JS-K, the role of GSTs in liberating NO from JS-K has been amply and repeatedly demonstrated in numerous studies. Increased loads of enzyme gave the expected enhanced NO production from the prodrug^6^. However, confusion about the identity of the effective members of the GSTome still exists. For example, a study of HepG2 cells^15^ reports that GST P1-1 was catalyzing the release of NO from JS-K, in spite of the lack of detectable GST P1-1 protein in this cell line^16^. The majority of the GSTs involved in drug metabolism have not been tested with JS-K as a substrate, even though assays with three human enzymes representing the alpha, mu, and pi classes have been published^8, 17^. A more complete investigation of relevant human GSTs is required for understanding the scope of JS-K biotransformation and the consequent cellular effects in neoplastic as well as normal tissues. In the present study the majority of the members of the human GSTome^18^ is tested for activity with JS-K, and their suggested role in the antitumoral activity of the prodrug discussed.

## Materials and methods

JS-K, glutathione (GSH), 1-chloro-2,4-dinitrobenzene (CDNB) and all other biochemicals were purchased from Sigma-Aldrich. Expression clones of human GSTs were available in the laboratory as previously described^19^. The enzymes were purified on different occasions using various procedures; differences in the composition of the media containing stock solutions of the purified GSTs were negligible when the enzymes were diluted in the buffer of the JS-K assay.

### Protein expression

Colonies of *Escherichia coli* BL21 (DE3) harboring expression clones encoding recombinant GSTs M4-4, P1-1 and S1-1 were grown overnight in LB medium with 100 µg ml^−1^ ampicillin, in a shaking incubator at 200 rpm, 37 °C. The culture was diluted 100-fold and grown to A_600_ = 0.4, before induction with isopropyl-β-d-thiogalactoside (IPTG) to a final concentration of 0.2 mM. After 3 h the cells were harvested by centrifugation for 15 min at 7000*g*.

Bacterial cells containing GST M5-5 were cultivated in the manner described above with the exception that they were grown overnight without addition of an inducer.

Single colonies of *E. coli* XL1-blue expressing GSTs A1-1, A2-2, A3-3, M1-1, and M2-2 were grown overnight with shaking in 2TY medium containing 100 µg ml^−1^ ampicillin at 37 °C. Overnight cultures were then diluted 100-fold into 2TY containing 100 µg ml^−1^ ampicillin and allowed to grow at 37 °C with shaking until reaching exponential growth. Expression was then induced by addition of 0.2 mM IPTG and the cells were further grown overnight. Cells were harvested by centrifugation at 5000*g* for 5 min and the bacterial pellet was resuspended in 0.1 M sodium phosphate buffer, pH 7.0, containing 0.02% (w/v) sodium azide.

### Protein purification

Harvested bacterial cells were lysed on ice for 60 min by treatment with lysozyme (0.2 mg ml^−1^) and Protease Inhibitor Cocktail Tablets, EDTA-Free (Sigma-Aldrich) followed by sonication 5X 20 s and centrifuged at 30,000*g* for 30 min. All subsequent procedures were carried out at 4 °C.

Proteins with an N-terminal his tag (GSTs A1-1, A2-2, A3-3, P1-1, and S1-1) were purified as follows. The bacterial cells were harvested, resuspended in binding buffer (20 mM sodium phosphate, pH 7.4, containing 500 mM NaCl and 20 mM imidazole), and lysed by treatment as above, followed by sonication. After centrifugation at 30,000*g* for 30 min, the supernatant fraction was loaded onto a nickel–immobilized metal ion affinity chromatography (Ni–IMAC) column (His GraviTrap, GE Healthcare). Unbound protein was washed out with 10 ml of binding buffer. The recombinant GSTs were eluted with 3 ml of elution buffer containing 500 mM imidazole. The purified enzyme was dialyzed against 100 mM sodium phosphate buffer (pH 7.4). GST T1-1 was also his tagged and was purified as previous reported^20^. It should be noted that previous comparisons of his-tagged and untagged GSTs have not detected any significant differences caused by the tag in the catalytic properties of the enzymes.

Proteins without his tag were purified by affinity chromatography either on glutathione^21^ (GSTs A4-4, M1-1, M2-2 and M5-5) or on S-hexyl-glutathione^22^ (GSTM4-4) linked to epoxy-activated Sepharose 6B (GE Healthcare). The procedure was as follows: equilibration of the affinity gel as a batch using a glass filter funnel with binding/washing buffer consisting of 10 mM Tris–HCl pH 7.8, 0.2 M NaCl, 0.2 mM dithiothreitol (DTT) and 1 mM EDTA. Binding of the GST to the affinity gel by adding the lysate and incubating for 1 h at 4 °C on a tilting board. The affinity gel was washed repeatedly with the same buffer before elution of the protein with 20 mM GSH or 20 mM S-hexyl-glutathione in binding/washing buffer. The eluted GST was dialyzed overnight against 10 mM Tris–HCl pH 7.8, 0.2 mM DTT, 1 mM EDTA and 0.02% (w/v) sodium azide.

The protein content was determined with the Bio-Rad protein assay. The purity of the dialyzed protein was checked with SDS-PAGE.

### Standard assays of GST activity

The activities of the GST stocks were assayed at 30 °C with the standard substrates, 1 mM CDNB (stock solution in ethanol) and 1 mM GSH in 0.1 M sodium phosphate buffer pH 6.5, containing 1 mM EDTA, and the formation of the product S-2,4-dinitrophenyl-glutathione (DNP-SG) was followed spectrophotometrically at 340 nm^23^. The measured activities were compared with formerly determined specific activity values^19^ in order to assess the concentration of active enzyme in cases of divergence between the values.

GSTs activities with the prodrug were measured at 30 °C with 50 µM JS-K and 1 mM GSH in10 mM Tris–HCl pH 7.5, 1 mM EDTA and 0.1% (w/v) bovine serum albumin (BSA). The JS-K stock solution was made in acetonitrile and the concentration of the solvent in the enzyme assay was 5% (v/v). The reaction was monitored by DNP-SG formation followed spectrophotometrically at 340 nm, as the product is the same as with CDNB above. All measurements were performed on a Multiscan GO spectrophotometer (Thermo Fisher).

### Michaelis–Menten kinetics

Initial rate measurements with varied concentration of JS-K at constant 1 mM GSH were carried out at 30 °C in 10 mM Tris–HCl pH 7.5, 1 mM EDTA and 0.1% (w/v) BSA. Because of the limited solubility of JS-K in aqueous media no concentration higher than 50 µM was used in the measurements. The enzyme amount used in the 100 µl reaction system was 32 ng with GST A1-1 in Fig. 3a and 30 ng with GST M2-2 in Fig. 3b. The data were corrected for the non-enzymatic reaction rates and analyzed by non-linear regression using GraphPad Prism 8 software https://www.graphpad.com/scientific-software/prism/.

## Results and discussion

**Figure 2:**
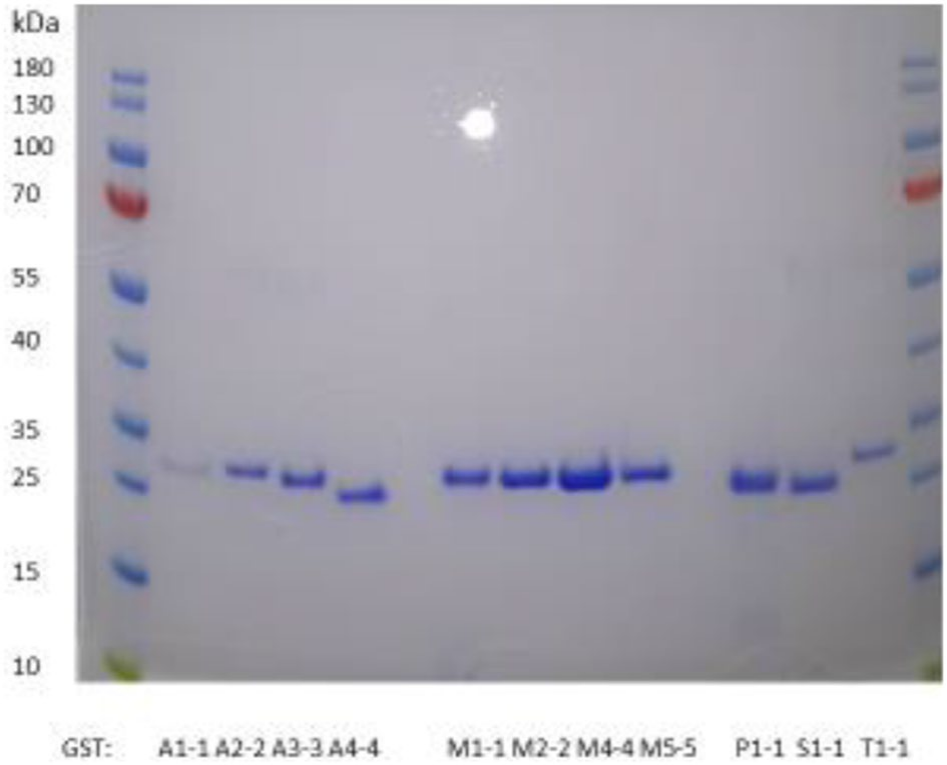
Analysis by SDS-PAGE of purified human GSTs stained with Coomassie Brilliant Blue.

A spectrophotometric assay of the reaction of glutathione with JS-K (Fig. 1) was based on the formation of DNP-SG measurable at 340 nm, as in the reaction with 1-chloro-2,4-dinitrobenzene (CDNB) described in 1967 and is commonly used to monitor GST activity^24^. The leaving group in the conjugation of JS-K is 1-[(4-ethoxycarbonyl)piperazin-1-yl]diazen-1-ium-1,2-diolate, which rapidly decomposes into 4-ethoxycarbonylpiperazine and the projected two active molecules of NO^8^. The human genome encodes up to 17 genes of cytosolic (soluble) GSTs, but two of them are frequently represented by null alleles, GSTM1*0 and GSTT1*0, such that the affected individuals do not express the corresponding enzymes^9^. Five of the seven classes of cytosolic GSTs are primarily responsible for detoxication of drugs and other xenobiotics: alpha (A), mu (M), pi (P), sigma (S), and theta (T). In the present investigation the classes were represented by human GST A1-1, GST A2-2, GST A3-3, GST A4-4, GST M1-1, GST M2-2, GST M4-4, GST M5-5, GST P1-1, GST S1-1, and GST T1-1. All enzymes were obtained as recombinant proteins and were demonstrated to be pure by SDS-PAGE (Fig. 2).

Table 1 shows the activities with JS-K expressed per mg GST protein. For comparison, the specific activities of the enzymes with CDNB as substrate are given. All enzymes tested except GST T1-1 demonstrated measurable activity with JS-K. Notably, human GST T1-1 also has negligible catalytic activity with CDNB^20^. The JS-K activities of the various GSTs span a > 100-fold range.

**Table 1:** Specific activities of human GSTs with JS-K and CDNB.

GST	JS-K	CDNB
	µmol min^−1^ mg^−1^	µmol min^−1^ mg^−1^
A1-1	42.5 ± 3.5	82
A2-2	37.2 ± 0.6	80
A3-3	18.3 ± 0.5	23
A4-4	6.3 ± 0.2	7.5
M1-1	57.8 ± 2.3	180
M2-2	273.0 ± 4.7	400
M4-4	0.89 ± 0.02	1.4
M5-5	58.0 ± 0.6	170
P1-1	1.2 ± 0.05	100
S1-1	12.9 ± 0.01	4
T1-1	Nd	0.005

Assays were made spectrophotometrically at 30 °C and pH 7.5 with 50 µM JS-K and 1 mM GSH. All measurements were made in triplicate and values are means, corrected for background reaction, ± S.D. (standard deviation); Nd (no detectable activity). The CDNB activities (at pH 6.5) have previously been reported^19^.

GST M2-2 displayed the highest specific activity of the tested enzymes: 273 µmol min^−1^ mg^−1^ (Table 1). Two other mu class members, GST M5-5 and the polymorphic GST M1-1, gave specific activities of 58 and 57.8 µmol min^−1^ mg^−1^, respectively. GST M4-4, which generally has low catalytic activities with various substrates^25^, showed the lowest value of all in the mu class.

Among the alpha class members, GST A1-1 had the highest specific activity: 42.5 µmol min^−1^ mg^−1^ followed by GST A2-2 and GST A3-3 with 37.2 and 18.3 µmol min^−1^ mg^−1^, respectively. The lowest activity in the alpha class was shown by GST A4-4, which is also known to have modest CDNB activity^26^.

The sigma class enzyme GST S1-1 presented a specific activity of 12.9, twice the GST A4-4 value of 6.3 µmol min^−1^ mg^−1^, and likewise has low CDNB activity^27^. GST S1-1 is distinguished as the hematopoietic prostaglandin D_2_ synthase (HPGDS).

The pi class member GST P1-1 ranks among the least active GSTs with a specific activity of 1.2 µmol min^−1^ mg^−1^, which is noteworthy, because this enzyme is present in most tissues^19^ (but not in the hepatocytes of the liver^28^). In some tumors GST P1-1 is overexpressed^11, 12^.

**Figure 3:**
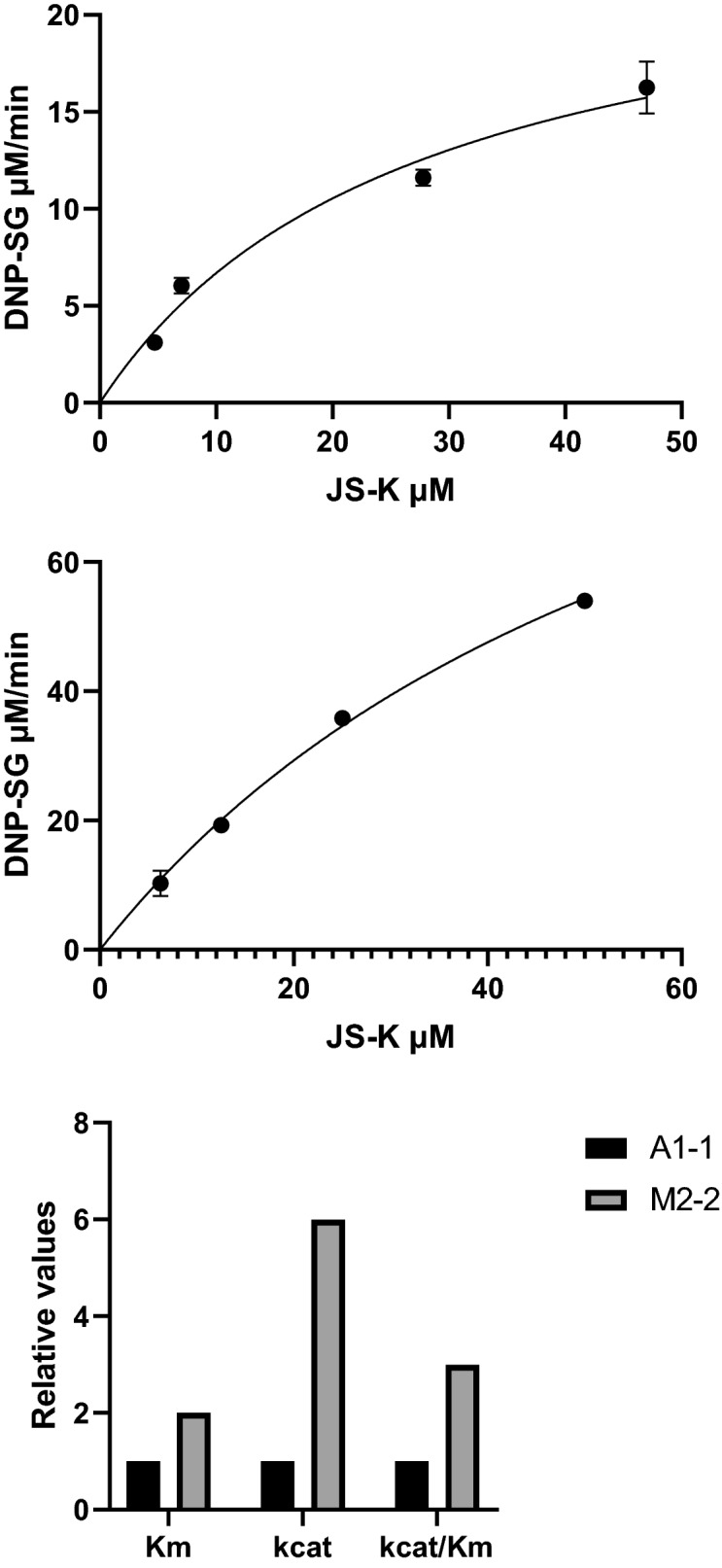
(**a**) Substrate saturation curve of human GST A1-1 with JS-K. (**b**) Substrate saturation curve of human GST M2-2 with JS-K. The assay was performed spectrophotometrically at 30 °C and pH 7.5 with 1 mM GSH and varied JS-K concentrations. Data points are represented as means and standard deviation of triplicate measurements. The Michaelis–Menten equation was fitted to the experimental data by nonlinear regression using Graphpad Prism 8 software. (**c**) Comparison of steady-state kinetic parameters in the conjugation JS-K by human GST A1-1 and GST M2-2. Relative values are shown normalized against the parameters of GST A1-1. Original values were: GSTA 1–1: Km 27 ± 6.2 µM, kcat 55 ± 6.2 s^−1^, kcat/Km 2 × 10^6^ M^−1^ s^−1^; GST M2-2: Km 63 ± 4 µM, kcat 353 ± 4 s^−1^ kcat/Km 6 × 10^6^ M^−1^ s^−1^.

Specific activities have previously been reported for commercial enzyme preparations of GST M1-1 (3.7 µmol min^−1^ mg^−1^), GST A1-1 (14.9 µmol min^−1^ mg^−1^), and GST P1-1 (0.15 µmol min^−1^ mg^−1^) at pH 6.5 and 25 °C^8^, but later redetermined with somewhat altered conditions of pH 7.4 and 37 °C to the higher values of 66.3, 126, and 1.36 µmol min^−1^ mg^−1^, respectively^17^ The latter values are in general agreement with the values in Table 1 considering differences in reaction temperature and other conditions. On the other hand, the catalytic efficiency k_cat_/Km of 20 mM^−1^ s^−1^ for GST A1-1 reported by the same group is two orders of magnitude lower than our value of 2000 mM^−1^ s^−1^ in Fig. 3c. Our value of 6000 mM^−1^ s^−1^ for GST M2-2 is even higher. The catalytic efficiency of mu class GST M1-1 was reported earlier as 62 mM^−1^ s^−1^^17^.

**Figure 4:**
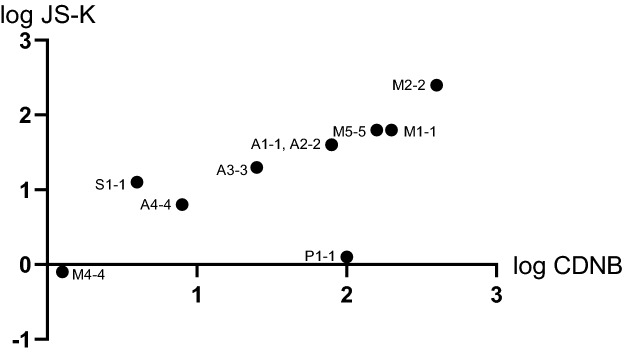
Correlation between specific activities of human GSTs with JS-K and CDNB as substrates. The data point for GST P1-1, with low JS-K and high CDNB activity, is a prominent outlier not included in the correlation analysis, which yields a slope of 1.0 and R^2^ = 0.96.

In general, the activities with JS-K were highly correlated with the activities with CDNB (Fig. 4). This relationship can be rationalized by the similar chemical mechanism, involving an aromatic nucleophilic attack by the sulfur of glutathione to form a Meisenheimer σ-complex, which is followed by the release of the common conjugate S-2,4-dinitrophenylglutathione. A notable exception was the low JS-K activity of GST P1-1, which contrasts with the high CDNB activity of the same enzyme. Apparently, the second product moiety of JS-K suppresses the release of S-2,4-dinitrophenylglutathione. In an opposite manner, GST S1-1 deviates somewhat from the general correlation, by being somewhat more active with JS-K than with CDNB (Fig. 4).

The antiproliferative property of JS-K was originally assumed to be based solely on NO released from the prodrug as the effective agent^8^. An alternative possibility involving S-2,4-dinitrophenylation of thiols or other cellular nucleophiles was excluded based on the lack of an equal effect of CDNB, which could produce a similar arylation without release of NO. However, the finding that pretreatment of HL-60 human myeloid leukemia cells with buthionine sulfoxide, an inhibitor of glutathione biosynthesis, did not suppress the antineoplastic effect of JS-K suggests that mechanisms other than the glutathione-activated NO discharge contribute^8^. Accumulating evidence points to the role of signaling through mitogen-activated protein kinases, cell factor β-catenin/T, and ubiquitin–proteasome pathways in cancer cell apoptosis^29^. Even though the molecular mechanisms underlying the therapeutic potential of JS-K remain unresolved, GSTs are definitely potent activators of NO release from the compound as also demonstrated in numerous prior studies. Our investigation has now revealed the scope of the GSTome in the biotransformation of JS-K. The specific activity of 273 µmol min^−1^ mg^−1^ displayed by GST M2-2 falls in the domain of the highest activities with any of a wide variety of alternative chemical reactions catalyzed by this enzyme^30^. The catalytic efficiency k_cat_/K_m_ of 6 × 10^6^ M^−1^ s^−1^ is approaching the values of the most efficient enzymes^31^. Two additional mu class enzymes, GST M1-1 and GST M5-5, show 20% of the GST M2-2 activity, and the alpha class members GST A1-1 and GST A2-2 are almost as active (Table 1).

The GSTs as an ensemble of enzymes occur abundantly in all tissues^32–34^, and it would appear that the GSTs collectively provide the means for efficient metabolism of JS-K. GST M2-2 is not expressed in liver, but the highly active GSTs A1-1 and A2-2 and GST M1-1 (except in subjects genetically homozygously *GSTM1* null) are highly abundant hepatic enzymes such that the tissue has high capacity for the biotransformation of JS-K. Particularly high activity can be expected also in testis, small intestine, kidney, and adrenal gland, based on the high expression levels of the latter enzymes. By contrast, uterus reportedly expresses only GST P1-1 and GST T1-1, and like erythrocytes, therefore has comparatively low activity with JS-K.

Overall, it would appear that the abundance of GSTs and their predominantly high activity with JS-K would cause the rapid biotransformation of the prodrug in most tissues. Even though many tumors overexpress GST P1-1^11, 35, 36^ it is not obvious that the elevation is sufficient to compensate for the relatively low activity of this enzyme in comparison with other GSTs and make the neoplastic cells selectively vulnerable to release of NO from JS-K. In some tumors elevated levels of alpha and mu class GST have also been reported, but neither in these instances can the antineoplastic activity of JS-K be attributed exclusively to GST-mediated release of NO.

We conclude that the established antitumoral effect of JS-K involves mechanisms in addition to NO release, and that the interplay between them and the unmistakable degradation of JS-K catalyzed by GSTs requires further incisive investigation.
